# Development of a method to create uniform phantoms for task‐based assessment of CT image quality

**DOI:** 10.1002/acm2.12974

**Published:** 2020-07-28

**Authors:** Juliane Conzelmann, Felix Benjamin Schwarz, Bernd Hamm, Michael Scheel, Paul Jahnke

**Affiliations:** ^1^ Department of Radiology Charité – Universitätsmedizin Berlin, Corporate Member of Freie Universität Berlin Humboldt‐Universität zu Berlin Berlin Institute of Health Berlin Germany; ^2^ Department of Neuroradiology Charité – Universitätsmedizin Berlin, Corporate Member of Freie Universität Berlin Humboldt‐Universität zu Berlin Berlin Institute of Health Berlin Germany

**Keywords:** contrast sensitivity, phantoms, imaging, tomography, x‐ray computed

## Abstract

**Purpose:**

To develop a customized method to produce uniform phantoms for task‐based assessment of CT image quality.

**Methods:**

Contrasts between polymethyl methacrylate (PMMA) and fructose solutions of different concentrations (240, 250, 260, 280, 290, 300, 310, 320, 330, and 340 mg/mL) were calculated. A phantom was produced by laser cutting PMMA slabs to the shape of a patient’s neck. An opening of 10 mm diameter was cut into the left parapharyngeal space. An angioplasty balloon was inserted and filled with the fructose solutions to simulate low‐contrast lesions. The phantom was scanned with six tube currents. Images were reconstructed with filtered back projection (FBP) and adaptive iterative dose reduction 3D (AIDR 3D). Calculated and measured contrasts were compared. The phantom was evaluated in a detectability experiment using images with 4 and 20 HU lesion contrast.

**Results:**

Low‐contrast lesions of 4, 9, 11, 13, 18, 20, 24, 30, 35, and 37 HU contrast were simulated. Calculated and measured contrasts correlated excellently (r = 0.998; 95% confidence interval: 0.991 to 1). The mean ± SD difference was 0.41 ± 2.32 HU (*P* < 0.0001). Detection accuracy and reader confidence were 62.9 ± 18.2% and 1.58 ± 0.68 for 4 HU lesion contrast and 99.6 ± 1.3% and 4.27 ± 0.92 for 20 HU lesion contrast (*P* < 0.0001), confirming that the method produced lesions at the threshold of detectability.

**Conclusion:**

A cost‐effective and flexible approach was developed to create uniform phantoms with low‐contrast signals. The method should facilitate access to customized phantoms for task‐based image quality assessment.

## 1 INTRODUCTION

Task‐based methods assess image quality based on the premise that image quality should be assessed in terms of meeting the medical goal of an image, often referred to as the diagnostic task.^1^ A frequent approach is to test how well an observer is able to detect a low‐contrast signal, for which the ground truth of the signal’s presence and location needs to be known. Task‐based methods require phantoms with embedded low‐contrast signals, which are also known as low‐contrast detectability (LCD) phantoms.

Several LCD phantoms are commercially available and were previously used for task‐based image quality assessment.^2^, ^3^, ^4^ The ACR accreditation phantom (Gammex, Middleton, WI) contains cylinders of 2 to 25 mm diameter and 6 HU contrast. The phantom can be expanded with the Advanced iqModule, which has cylinders of 1.5 to 25 mm diameter and 3, 6, or 10 HU contrast. The Catphan phantom (Phantom Laboratories, Salem, NY) has a module with a series of cylindrical rods of 2 to 15 mm diameter and 0.3 to 1% contrast. The same company also provides the MITA IQ low‐contrast phantom, which has rods of 3 to 15 mm diameter and 3 to 14 HU contrast.

As these phantoms differ in their arrangement of signals, signal diameters, and contrasts, they can offer advantages for different experimental designs, for example with regard to the choice of signals at the interface between detectable and undetectable. However, most institutions do not have all of these phantoms at their disposal. Furthermore, some requirements are not fulfilled by any of the commercially available phantoms, for example, large signal spacing and multiple signals at the threshold of detectability.^5^ Previous work used customized LCD phantoms that were tailored to a specific experimental setup.^6^ However, such phantoms often have to be ordered from specialized manufacturers, which can delay studies and cause significant costs. A fast, cost‐effective, and flexible method would be desirable to facilitate access to LCD phantoms which could ideally be tailored to particular study designs.

To create phantoms with low‐contrast signals, at least two different materials must be combined. The contrast results from the difference of these materials’ linear attenuation coefficients and can be calculated, if the chemical composition and the physical density of the materials are known. Polymethyl methacrylate (PMMA) is a uniform material frequently used for phantom bodies.^7^, ^8^ A second material to generate a signal can be a homogeneous fructose solution, which has the advantage that the contrast can be adjusted by the concentration of the solution. The present study explored these materials for the construction of LCD phantoms. The hypothesis was that calculated contrasts between two materials can be used as a basis to produce a uniform phantom with corresponding low‐contrast signals. As part of ongoing work to evaluate a CT system for neck imaging, a neck‐shaped phantom was created as proof of principle to illustrate the approach. The aim was to develop a customized method to produce uniform phantoms for task‐based assessment of CT image quality.

## 2 MATERIALS AND METHODS

The institutional ethics committee approved the study and waived informed consent.

### 2.A. Calculation of Hounsfield units and contrast values

Mass attenuation coefficients of PMMA, water, and aqueous fructose solutions (240, 250, 260, 280, 290, 300, 310, 320, 330, and 340 mg/mL) were calculated as weighted sums of the mass attenuation coefficients of their atomic constituents [Eq. (1)] as provided by the National Institute of Standards and Technology (NIST) database.^9^ The calculations were performed in the same way for PMMA, water, and fructose solutions.

(1) $$\frac{\mu }{\rho }=\sum w_{i}{\left(\frac{\mu }{\rho }\right)}_{i}$$

Equation (1), where $\frac{\mu }{\rho }$ is the mass attenuation coefficient (cm^2^/g) and $w_{i}$ is the mass fraction of element i.

Mass attenuation coefficients were used at 72 keV based on preliminary experiments to approximate the mean photon energy at 120 kVp CT imaging. The physical density of PMMA was provided by the manufacturer (1.18 g/cm^3^). Densities of fructose solutions at 20°C were obtained from interpolation of published data.^10^ Linear attenuation coefficients were calculated by multiplying the mass attenuation coefficients of PMMA, water, and the fructose solutions with their respective physical density. Hounsfield units (HU) were calculated, and contrast values were calculated as difference between PMMA HU and fructose HU.

### 2.B. Phantom construction

**Fig. 1:**
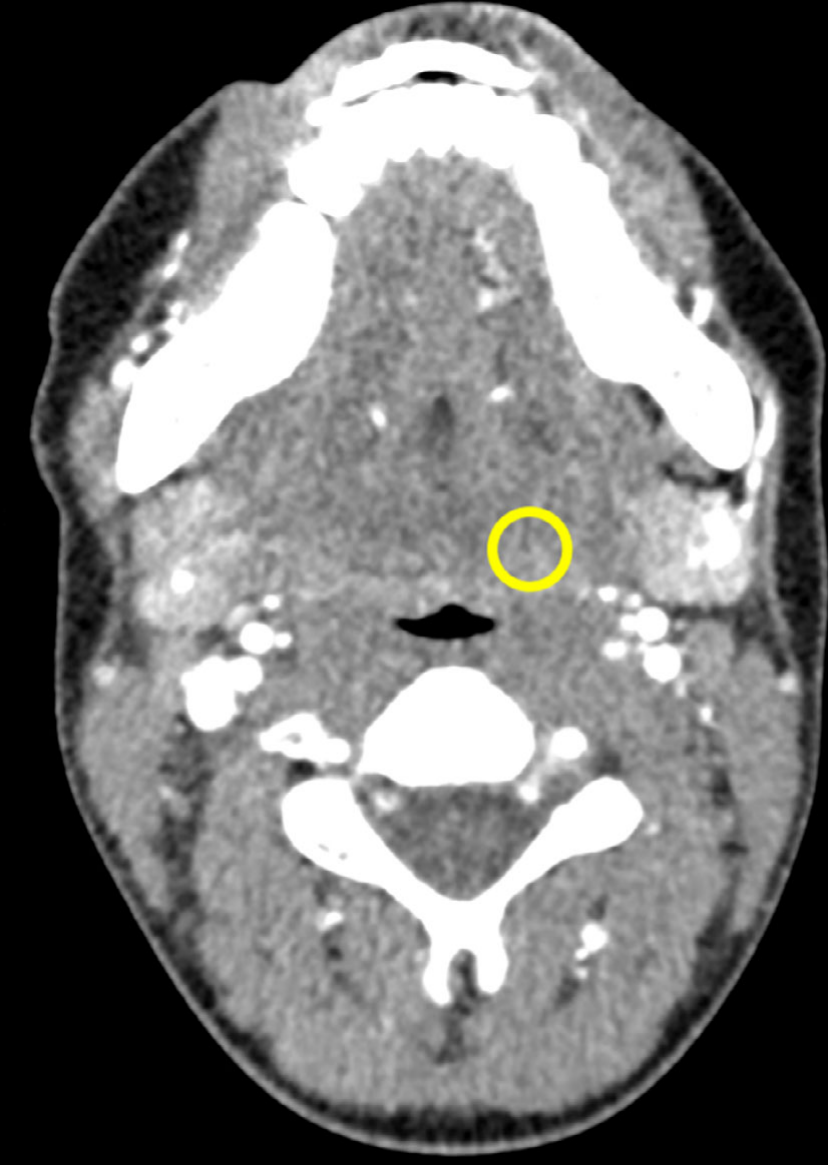
Neck computed tomography image of a patient that was used as a template for the phantom shape. The region of interest in the left parapharyngeal space indicates the position for insertion of low‐contrast signals.

A neck CT image of a patient was used as template to create a phantom with the patient’s neck shape. The dimensions were 15.4 cm (length) × 10.6 cm (width). A circular area of 10 mm diameter in the left parapharyngeal space was selected to insert low‐contrast signals. Figure 1 shows the CT image of the patient and the signal position. Nineteen PMMA slabs of 5 mm thickness were laser cut to the patient's shape. Circular openings of 10 mm diameter were inserted into four slabs and openings of 14 mm diameter were inserted into 11 slabs in the left parapharyngeal space. The remaining four slabs did not contain any openings. Next, the metal markers were removed from a Mustang angioplasty balloon of 12 mm diameter and 4 cm length (Boston Scientific, Marlborough, MA) to avoid artifacts that could interfere with HU measurement and detection tasks: First, the balloon was opened at the top and the markers were removed. Second, the balloon was closed with thin thread and sealed with cyanoacrylate. After these preparations, the PMMA slabs were stacked so that slabs with 10 and 14 mm openings were stacked alternately. The slabs were compressed between two wood panels, the angioplasty balloon was inserted into the opening and filled with the fructose solutions. Alternating stacking of PMMA slabs with 10 and 14 mm openings was done to prevent small offsets due to imperfect stacking, which might lead to air artifacts resulting from incomplete expansion of the balloon to the wall of the 10 mm opening. For the subsequent analysis of lesion contrasts and detectability, only images of the phantom part with 10 mm openings were used.

### 2.C. CT image acquisition

The phantom was examined with a Canon Aquilion Prime CT (Canon Medical Systems, Otawara, Japan). All acquisitions were performed in helical mode with a fixed collimation of 80 × 0.5 mm, rotation time of 0.5 s, pitch of 0.813, and M‐size field of view (FOV) of 280 mm diameter. These are the standard settings used for neck imaging on our CT system. The tube voltage was 120 kVp. Six tube currents were used: 10, 20, 30, 40, 100, and 120 mA. CTDIvol values were 0.5, 0.9, 1.4, 1.9, 4.7, and 5.6 mGy, respectively. Images were reconstructed with filtered back projection (FBP) and adaptive iterative dose reduction 3D (AIDR 3D). The standard soft tissue kernel (FC08) and 0.5 mm thin slice reconstruction as in our clinical neck protocol were used. Five repeated acquisitions were performed per tube current and fructose concentration. A total of 600 data sets were generated (10 fructose concentrations × 6 tube currents × 2 reconstruction methods × 5 repetitions).

### 2.D. HU and contrast analysis

Nine images per data set were used to measure contrasts between low‐contrast lesions simulated with the fructose solutions and the PMMA background. On every image, one circular region of interest (ROI) of 8 mm diameter was placed into the low‐contrast lesion and six circular ROIs of 25 mm diameter were placed into the PMMA background surrounding the lesion. The contrast per image was calculated as the difference between mean PMMA HU and fructose HU. For comparison with calculated contrasts, the measured contrast values were averaged over all data sets to reduce bias resulting from effects of acquisition and reconstruction parameters on measured values.^11^, ^12^ In addition to the CT scans of the neck phantom, we also analyzed HU in CT images of a 16‐cm CTDI phantom, which, like the background of the neck phantom developed in our study, consists of PMMA. CT images of the CTDI phantom were acquired in the same manner as for the neck phantom (6 tube currents × 2 reconstructions × 5 repetitions), and HU measurements were also analyzed in the same way for six circular ROIs of 25 mm diameter in nine images per acquisition.

### 2.E. Detectability experiment

A detectability experiment was performed to investigate whether the neck phantom actually yields the expected decrease in detectability and reader confidence at low lesion contrast as opposed to relatively high lesion contrast. The experiment was performed to verify that the method of phantom construction did not introduce signals or artifacts (such as transition artifacts between the balloon and the PMMA phantom body) possibly resulting in similar detectability of low and high contrast signals, which would compromise the purpose of the phantom. To that end, a four‐alternative forced choice experiment with seven blinded radiologists was performed using data sets acquired with 290 and 340 mg/mL fructose concentration, 30 and 120 mA tube current, and reconstructed with FBP and AIDR 3D. Four lesion images and 12 nonlesion images per acquisition were extracted. In total, 160 lesion images (2 fructose concentrations × 2 tube currents × 2 reconstruction methods × 5 repeated acquisitions × 4 images) and 480 nonlesion images were extracted. Every lesion image was paired with three nonlesion images acquired with identical acquisition and reconstruction properties and presented to the readers. Readers were asked to select the image containing a lesion and to indicate their confidence on a five‐step scale from 1 = not confident to 5 = confident. Readings were performed using in‐house developed software on diagnostic screens (Eizo RadiForce RX250, Eizo Corporation, Hakusan, Japan) in reading rooms with ambient light conditions.

### 2.F. Data analysis

Measured HU values are presented as mean ± standard deviation (SD) and as median and range. Correlation analysis was performed using Pearson correlation. Estimates are given as correlation coefficient *r* and 95% confidence intervals (CI). Detection accuracy was calculated as the percentage of correct lesion selections per reader. Calculated and measured contrast values as well as detection accuracy and reader confidence at 4 and 20 HU signal contrast were compared with t‐tests. Effects of dose and image reconstruction methods on detectability were compared with analysis of variance for repeated measurements by using post‐hoc tests with Tukey's method to adjust for multiple comparisons. Differences were interpreted as significant when *P* < 0.05.

## 3 RESULTS

### 3.A. Phantom

**Fig. 2:**
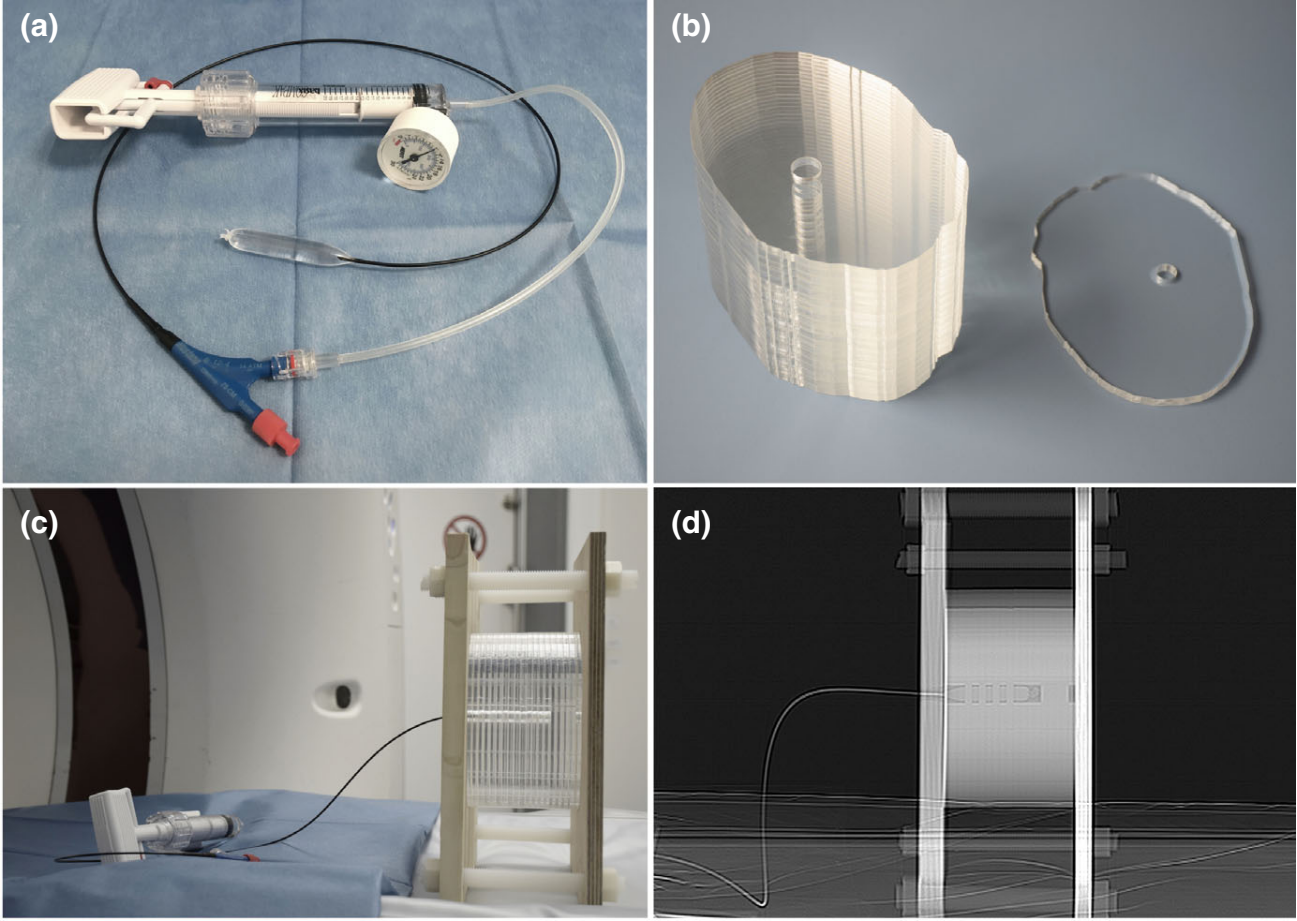
Phantom construction. (a) The metallic markers were removed from a 12 mm diameter angioplasty balloon, which was filled with fructose solutions. (b) Nineteen PMMA slabs of 5 mm thickness were cut to the shape of a patient's neck and an opening was inserted into the left parapharyngeal space to hold the balloon. (c) The PMMA slabs were compressed between two wood panels and the angioplasty balloon was inserted. (d) Computed tomography scout image of the phantom holding the angioplasty balloon.

**Fig. 3:**
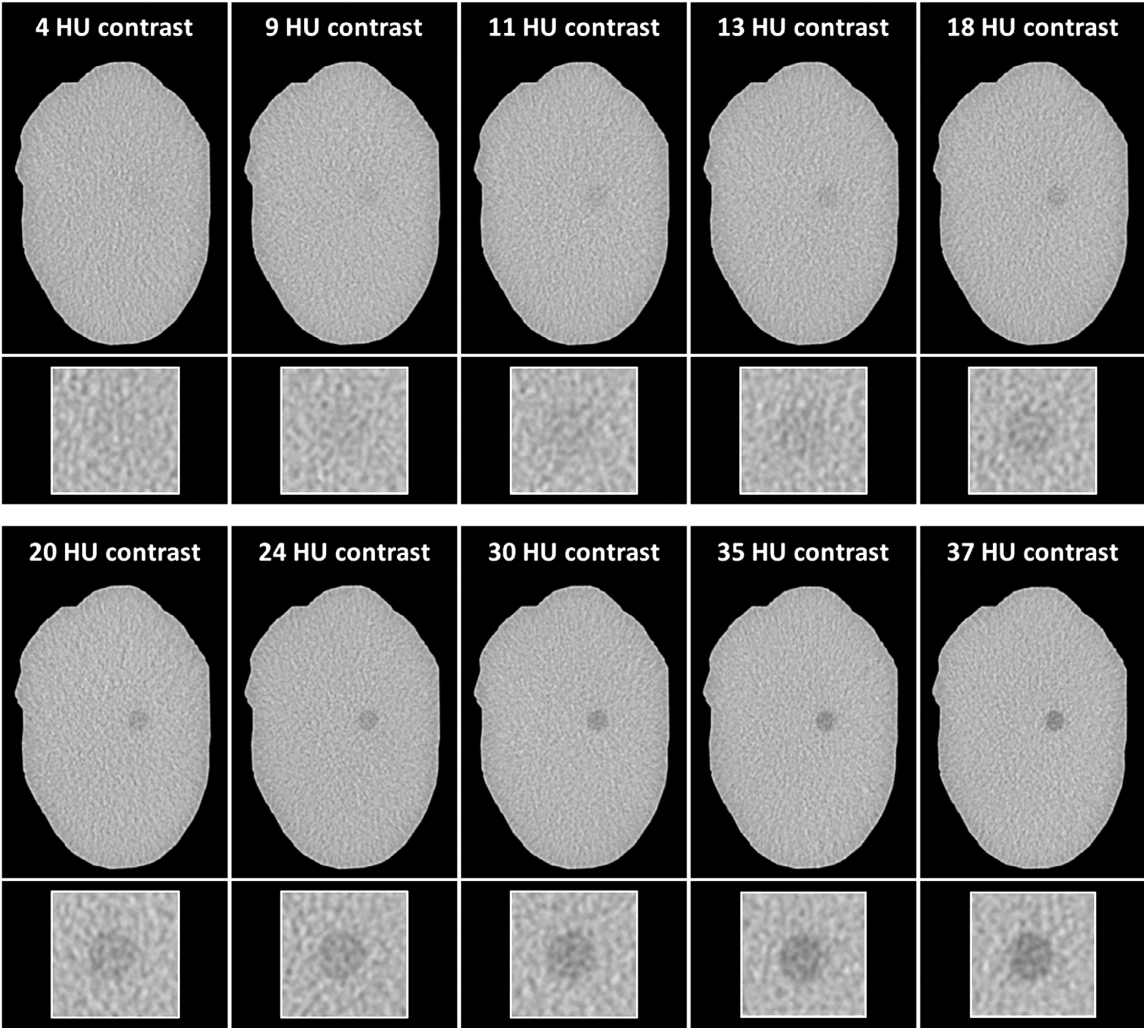
Computed tomography images of the phantom with different fructose concentrations. Displayed images were acquired with 120 mA tube current (CTDIvol 5.6 mGy) and reconstructed with FBP. Labels at the top indicate measured contrast values. The bottom row shows magnified details of the lesions in the left parapharyngeal space. All images are displayed with window level 100 and window width 350.

Figure 2 shows photographs of the fructose‐filled angioplasty balloon, the PMMA phantom, the phantom setup in the CT scanner, and a scout image of the phantom. Figure 3 shows CT images of the phantom with low‐contrast lesions generated with fructose solutions of different concentrations.

### 3.B. HU and contrast analysis

**Fig. 4:**
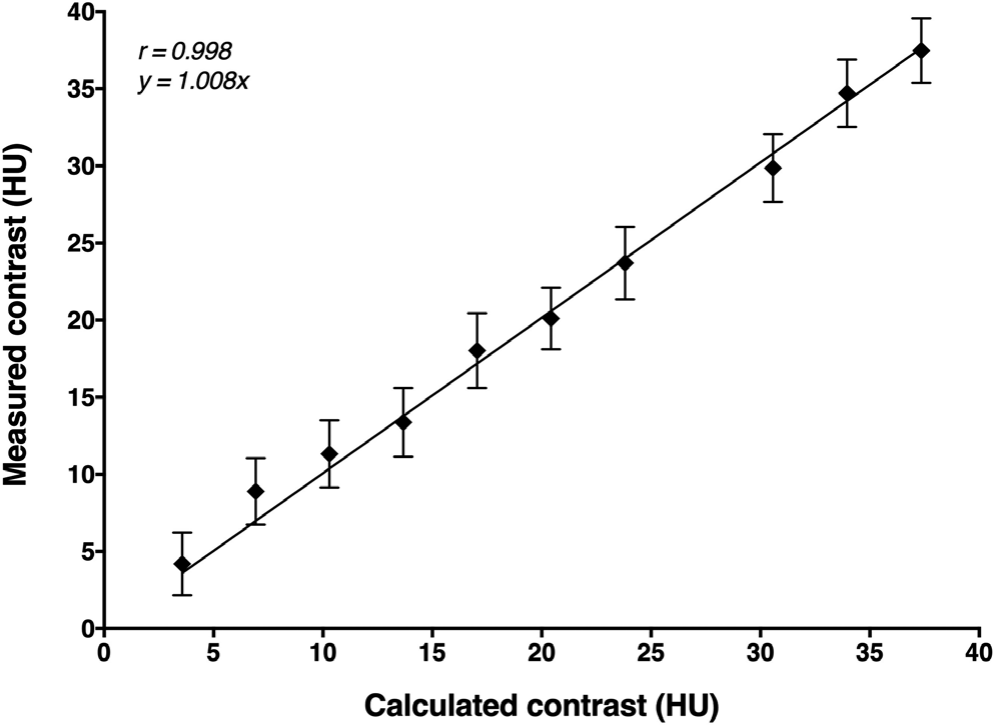
Calculated and measured lesion contrast values. Means and standard deviations across all acquisitions and reconstructions, Pearson's correlation coefficient *r* and the equation of the linear regression are shown.

Measured contrast values correlated excellently with calculated values (Fig. 4). Across all acquisitions and image reconstructions, Pearson’s correlation coefficient *r * was 0.998 (95 % confidence interval: 0.991 to 1). The equation of the linear regression was y = 1.008x. The mean ± SD difference between measured and calculated contrast values across all 5,400 analyzed images was 0.41 ± 2.32 HU (*P* < 0.0001). The median across all ten fructose concentrations was 0.45, the range was −0.6 to 2. The calculated PMMA HU value was 118.6. Table 1 provides measured HU and contrast values averaged across all acquisitions and reconstructions in comparison with calculated values for all fructose concentrations. Table 2 additionally provides measured contrast values per tube current and image reconstruction separately and shows that contrast values varied slightly with different scan settings. The analysis of the 16‐cm CTDI phantom yielded a mean ± SD HU of 119.9 ± 1.0.

**Table 1:** Calculated and measured HU and contrast values. Mean ± SD of measured values averaged across all acquisitions and reconstructions are shown. Fructose HU and contrasts were calculated according to Methods Part A.

Fructose concentration (mg/mL)	Density (g/cm^3^)	Calculated fructose HU	Measured fructose HU	Measured PMMA HU	Calculated contrast	Measured contrast
240	1.090	81.3	80.8 ± 2.0	118.3 ± 0.8	37.3	37.5 ± 2.1
250	1.094	84.7	83.5 ± 2.1	118.2 ± 0.8	33.9	34.7 ± 2.2
260	1.097	88.1	87.6 ± 2.1	117.4 ± 1.1	30.5	29.9 ± 2.2
280	1.105	94.8	93.7 ± 2.3	117.4 ± 1.1	23.8	23.7 ± 2.4
290	1.109	98.2	97.2 ± 2.0	117.4 ± 1.1	20.4	20.1 ± 2.0
300	1.112	101.6	99.4 ± 2.4	117.4 ± 1.2	17	18.0 ± 2.4
310	1.116	105.0	103.9 ± 2.3	117.3 ± 1.2	13.6	13.4 ± 2.2
320	1.120	108.3	106.1 ± 2.2	117.5 ± 1.1	10.3	11.3 ± 2.2
330	1.124	111.7	108.5 ± 1.9	117.4 ± 1.2	6.9	8.9 ± 2.2
340	1.128	115.1	113.1 ± 2.1	117.3 ± 1.2	3.5	4.2 ± 2.0

**Table 2:** Measured contrast per fructose concentration, tube current, and reconstruction method. Mean ± SD values of 45 measurements are shown (five repeated acquisitions per condition × 9 images per acquisition).

Fructose concentration (mg/mL)	FBP						AIDR 3D					
	10 mA	20 mA	30 mA	40 mA	100 mA	120 mA	10 mA	20 mA	30 mA	40 mA	100 mA	120 mA
240	38.9 ± 4.5	37.7 ± 2.2	38.1 ± 1.9	37.0 ± 1.5	36.5 ± 0.9	36.4 ± 0.8	39.3 ± 2.2	37.6 ± 1.2	37.5 ± 1.2	37.0 ± 1.0	36.9 ± 0.8	36.7 ± 0.7
250	35.9 ± 4.7	34.8 ± 3.0	34.7 ± 2.2	34.8 ± 1.4	33.9 ± 0.9	33.5 ± 1.0	36.0 ± 2.1	35.1 ± 1.6	34.9 ± 1.6	34.7 ± 1.0	34.2 ± 0.8	33.8 ± 0.9
260	28.8 ± 4.2	30.8 ± 2.9	30.7 ± 1.9	29.6 ± 2.3	29.0 ± 1.0	29.1 ± 0.9	30.3 ± 2.6	30.7 ± 1.7	30.5 ± 1.4	29.9 ± 1.8	29.4 ± 0.9	29.5 ± 0.9
280	23.8 ± 5.2	24.1 ± 2.9	23.7 ± 2.5	23.6 ± 1.5	23.1 ± 1.2	22.7 ± 1.3	24.8 ± 2.4	24.6 ± 1.7	23.6 ± 2.0	23.9 ± 1.1	23.5 ± 1.1	23.2 ± 1.1
290	19.6 ± 4.1	21.2 ± 2.5	20.0 ± 2.2	19.7 ± 1.4	19.4 ± 1.0	19.3 ± 1.2	21.0 ± 1.6	21.5 ± 1.4	20.2 ± 1.6	20.0 ± 1.2	19.8 ± 0.9	19.7 ± 1.1
300	18.5 ± 5.9	18.7 ± 2.2	18.3 ± 2.3	16.8 ± 1.8	17.1 ± 1.3	17.3 ± 1.0	19.6 ± 2.5	18.8 ± 1.1	18.6 ± 1.6	17.5 ± 1.6	17.6 ± 1.2	17.8 ± 1.0
310	13.2 ± 5.5	13.7 ± 2.6	13.4 ± 2.4	12.6 ± 1.5	12.8 ± 1.2	12.7 ± 1.0	14.3 ± 1.7	14.2 ± 1.5	14.0 ± 1.4	13.2 ± 1.2	13.3 ± 1.2	13.2 ± 0.9
320	10.8 ± 4.5	12.1 ± 3.2	11.0 ± 2.1	10.8 ± 1.4	10.8 ± 1.1	10.3 ± 1.1	12.3 ± 2.2	12.4 ± 1.7	11.7 ± 1.5	11.5 ± 1.0	11.3 ± 1.0	10.8 ± 1.1
330	9.8 ± 4.3	9.0 ± 2.0	8.5 ± 2.4	8.1 ± 1.8	7.8 ± 1.6	8.0 ± 1.2	10.7 ± 1.6	9.8 ± 1.5	9.3 ± 1.7	8.8 ± 1.5	8.3 ± 1.5	8.6 ± 1.1
340	2.6 ± 3.6	4.4 ± 2.7	3.9 ± 2.2	3.4 ± 1.7	3.8 ± 1.2	3.8 ± 1.2	5.0 ± 1.7	5.7 ± 1.4	4.8 ± 1.7	4.4 ± 1.5	4.3 ± 1.1	4.3 ± 1.2

### 3.C. Detectability experiment

**Fig. 5:**
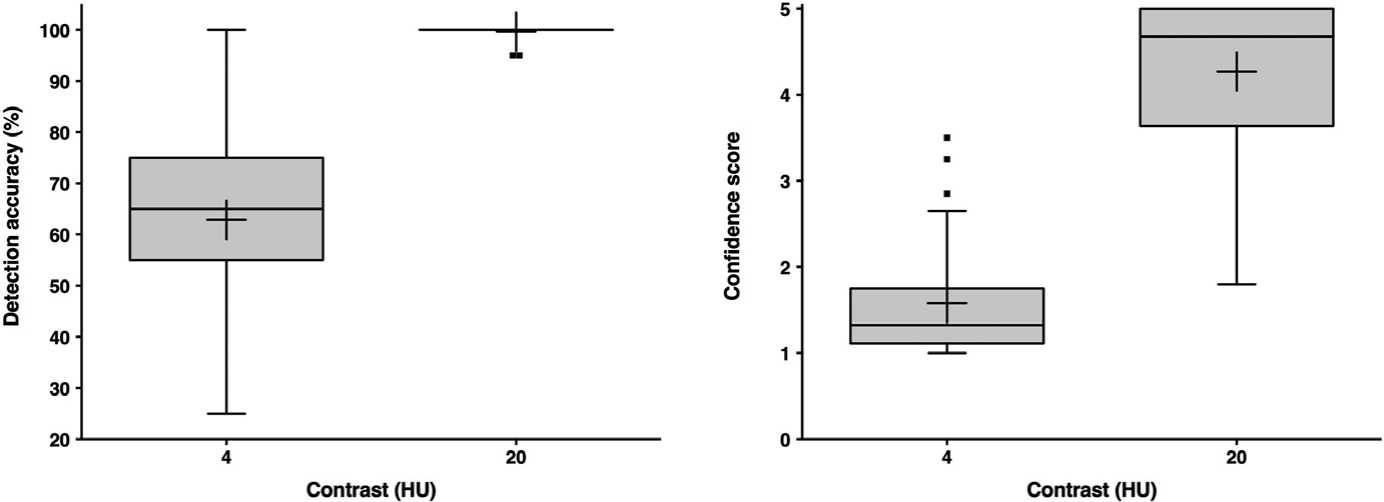
Comparison of detection accuracy (left) and confidence scores (right) between low and higher contrast signals. Pooled reader results across all tube currents and image reconstructions are shown. Crosses indicate mean values.

Figure 5 shows detection accuracy and confidence results for 4 and 20 HU signal contrast across all acquisitions and image reconstructions. Mean ± SD detection accuracy was 62.9 ± 18.2 % for 4 HU lesion contrast and increased to 99.6 ± 1.3 % for 20 HU contrast (*P* < 0.0001). Similarly, confidence values were low for 4 HU contrast (1.58 ± 0.68) and increased significantly to 4.27 ± 0.92 for 20 HU contrast (*P* < 0.0001). Table 3 provides separate detection accuracy results per tube current and image reconstruction. Table 4 summarizes corresponding confidence results. At 4 HU lesion contrast and 30 mA tube current (CTDIvol 1.4 mGy), detection accuracy was significantly higher for AIDR 3D‐than for FBP‐reconstructed images (*P* = 0.008), which was not the case at 120 mA tube current (CTDIvol 5.6 mGy) (*P* > 0.999). Detection accuracy significantly decreased between 120 and 30 mA tube current for FBP‐ (*P* = 0.0495), but not for AIDR 3D‐reconstructed images (*P* = 0.992).

**Table 3:** Detection accuracy per signal contrast, tube current, and reconstruction method. Mean ± SD values are shown. CTDIvol values were 1.4 mGy for 30 mA and 5.6 mGy for 120 mA tube current.

Signal contrast (HU)	30 mA FBP	30 mA AIDR 3D	120 mA FBP	120 mA AIDR 3D
4	42.9 ± 16.8 %	67.9 ± 10.8 %	70.0 ± 15.3 %	70.7 ± 15.1 %
20	98.6 ± 2.4 %	100.0 ± 0.0 %	100.0 ± 0.0 %	100.0 ± 0.0 %

**Table 4:** Reader confidence per signal contrast, tube current, and reconstruction method. Mean ± SD values are shown. CTDIvol values were 1.4 mGy for 30 mA and 5.6 mGy for 120 mA tube current.

Signal contrast (HU)	30 mA FBP	30 mA AIDR 3D	120 mA FBP	120 mA AIDR 3D
4	1.41 ± 0.58	1.59 ± 0.64	1.69 ± 0.84	1.63 ± 0.77
20	3.91 ± 1.19	4.26 ± 1.01	4.37 ± 0.76	4.54 ± 0.75

## 4 DISCUSSION

A frequent approach to task‐based image quality assessment is to test how well an image enables an observer to detect a signal, which requires to examine appropriate phantoms with embedded signals. The availability of such phantoms is limited and they cannot easily be tailored to specific study needs. The present study aimed at developing a method to produce customized phantoms for task‐based image quality assessment.

The development was based on the premise that two materials of different attenuation must be involved — one material for the phantom background (PMMA) and another material to generate signals of variable contrast (fructose solutions). Linear attenuation coefficients of these materials were calculated based on their known chemical composition and physical density. However, the mean CT energy spectrum was not known exactly and had to be approximated. Based on preliminary experiments, 72 keV were used for 120 kVp imaging, which was in good agreement with previous dual energy CT studies, where 120 kVp single energy images yielded similar HU values as 70–75 kVe monochromatic images.^13^, ^14^, ^15^, ^16^ Calculated HU values were in excellent agreement with measured values, confirming the validity of the calculation model. The observed slight variations in measured contrast values with different tube currents and image reconstructions were expected, as CT settings were previously shown to affect HU measurement.^11^, ^12^

The method presented here produced uniform signals, the contrast of which was simply adjusted by varying the fructose concentration. In the context of ongoing research in neck imaging, a neck‐shaped phantom was developed as proof of principle to illustrate the method. However, the technique of manufacturing the phantom can easily be adapted to create phantoms of different shapes for different research settings by varying the size and contour of the PMMA as well as the signal size by modifying the size of the balloon and PMMA openings. Furthermore, the technique can also be used to design a phantom that holds several balloons simultaneously, which would allow investigators to extract multiple ROIs per CT image for evaluating LCD. Another possible modification of the approach presented here could be to use a standard phantom body such as the CTDI phantom, which consists of PMMA and has five insert holes of 13.1 mm diameter. In the present study, HU of the CTDI phantom slightly differed from that of the PMMA background of our neck phantom, which is attributable to a higher physical density of the PMMA in the CTDI phantom (the physical density of PMMA can slightly vary around 1.18 g/cm^3^). Such effects can easily be determined by measuring CT numbers of the phantom body before preparing fructose solutions to simulate low‐contrast signals. An advantage of using a realistically shaped phantom body over the CTDI phantom might be that the asymmetrical shape may lead to different noise textures possibly affecting low‐contrast detectability.

The most important benefits of the method developed in this work are that it facilitates access to LCD phantoms and that it offers flexibility for preparing customized phantoms. Several LCD phantoms are commercially available such as the ACR accreditation phantom and the Advanced iqModule (Gammex, Middleton, WI), the Catphan phantom, and the MITA IQ low‐contrast phantom (Phantom Laboratories, Salem, NY). These phantoms differ in their arrangement, size, and contrasts of low‐contrast signals. For example, the ACR accreditation phantom only has rods of 6 HU contrast, whereas the Advanced iqModule additionally offers 3 and 10 HU contrast rods. In this phantom, rods of different sizes are grouped in close proximity, while the MITA IQ low‐contrast phantom contains fewer rods with wider spacing. Depending on the type of analysis (e.g., signal‐location‐known vs. signal‐location‐unknown experiments), it can be desirable to have as many signals as possible per CT image or to have wide spacing to extract different ROIs per CT image with variable signal positions. Also, the threshold of detectability is of particular relevance in LCD experiments. It can be desirable to have repeated versions of a certain signal size and contrast at the threshold of detectability per CT image to extract multiple ROIs containing that signal and avoid extensive scanning of the phantom.^5^ Not all of the possible requirements can be met by a single phantom, and some LCD scenarios are currently not addressed by any of the commercially available phantoms. In light of this situation, the method we present here provides a cost‐effective approach to the creation of LCD phantoms that can easily be modified to meet specific experimental needs and answer a range of different research questions.

The detectability experiment we performed ruled out signals or artifacts distorting the low‐contrast signals. Thus, our results show that the phantom we designed is suitable for its intended purpose. While detection accuracy and reader confidence were high at 20 HU lesion contrast, readers were unconfident in selecting the image containing the lesion, and their detection accuracy was low at 4 HU contrast. Results for 4 HU contrast were similar to those of a previous study investigating signals of 6 mm diameter and 5 HU contrast at similar dose levels.^17^ Analysis of results achieved with different tube currents and reconstruction methods showed dose reduction from 5.6 to 1.4 mGy to be feasible with AIDR 3D without compromising low‐contrast detectability but not with FBP. However, the initial detectability experiment we performed here aimed at evaluating the method for phantom construction and further investigations are needed to support conclusions regarding dose reduction or the choice of reconstruction technique. Future studies investigating CT techniques with the kind of phantom presented should expand the range of low signal contrasts in addition to the 4 HU contrast used here.

The limitations of this study include that only one lesion size was investigated and that long‐term stability and reproducibility were not investigated. HU values were only measured with one CT system, and results may slightly vary with different systems and energy spectra.^18^, ^19^ However, this limitation applies to all LCD phantoms, and such variations can be expected to be small, as only materials with low atomic numbers (and thus relatively low energy dependence) were used. Yet, if necessary, the calculation model could also be adapted to account for different energy spectra. Effects of dose and reconstruction methods were beyond the scope of this work and therefore not analyzed in detail. It should also be noted that a uniform phantom background texture was produced and that more complex textures were previously shown to affect detection results.^20^

In conclusion, the present work shows that contrast values between two uniform materials can be calculated and used to produce phantoms for task‐based image quality assessment. The method we propose facilitates access to such phantoms and provides flexibility in creating phantoms tailored to specific study designs involving detection tasks of low‐contrast signals for assessment of image quality.

## CONFLICT OF INTEREST

This study has received funding by the Bundesministerium für Wirtschaft und Energie (DE): 03EFHBE093. Three authors (F.B. Schwarz, M. Scheel, and P. Jahnke) are shareholders of a company that produces phantoms.
